# Wollastonite in Acrylic Paint to Protect Normal and Heat-Treated Spruce Wood Against *Coniophora puteana*

**DOI:** 10.3390/polym18070788

**Published:** 2026-03-25

**Authors:** Hamid R. Taghiyari, Elham Nadali, Antonio Pizzi, Afshin Rahmati, Olaf Schmidt, Antonios N. Papadopoulos

**Affiliations:** 1 Wood Science and Technology Department, Faculty of Civil Engineering, Shahid Rajaee Teacher Training University, Tehran 16788-15811, Iran; 2 Faculty of Natural Resources, Semnan University, Semnan 35131-19111, Iran; 3 Laboratoire d’Etude et Recherche sur le Matériau Bois (LERMAB), University of Lorraine, 27 Rue Philippe Seguin, 88000 Epinal, France; antonio.pizzi@univ-lorraine.fr; 4 Wood Biology, Institute of Wood Science, University of Hamburg, Leuschnerstr. 91d, 21031 Hamburg, Germany; 5 Department of Natural Environment and Climate Resilience, Democritus University of Thrace, 66100 Drama, Greece

**Keywords:** biological durability, compression parallel to grain, mass loss, mineral materials, thermal modification, wollastonite, wood-decay fungi

## Abstract

This study investigates the efficacy of wollastonite-enriched acrylic paint in protecting spruce wood (*Picea abies*) against the brown-rot fungus *Coniophora puteana*. Unheated and heat-treated wood samples (185 °C for 4 h) were coated with either plain acrylic paint or wollastonite-enriched acrylic paint and exposed to the fungus. Fungal resistance was evaluated by measuring mass loss (ML) and compression strength parallel to the grain. While conventional acrylic coatings provide a physical barrier against moisture and limited microbial attack, their effectiveness against *C. puteana* is often insufficient. Our results show that untreated controls lost 23.8% of their mass, whereas plain acrylic paint reduced mass loss only slightly. In contrast, wollastonite-enriched paint significantly decreased ML in both unheated and heat-treated specimens, demonstrating superior antifungal performance. These findings indicate that incorporating wollastonite into acrylic paint enhances fungal resistance, offering a simple, environmentally friendly, and effective surface treatment for spruce wood. This study fills a research gap in the use of mineral additives in acrylic coatings and highlights a practical approach for improving wood durability against fungal decay.

## 1. Introduction

The natural characteristics of wood make it an excellent material, but wood faces extreme danger because wood-decaying fungi attack its structure, which leads to fast deterioration and short lifespan in various uses. The brown-rot fungus *Coniophora puteana* stands out as one of the most dangerous wood-destroying fungi because it creates major damage to wood materials under high humidity conditions. The search for effective wood preservation methods has existed since the beginning of human civilization because ancient techniques used chemical and synthetic biocides for this purpose. The wood industry is experiencing rising demand for sustainable non-toxic solutions which has resulted in increased preference for environmentally friendly alternatives in recent years [1,2,3].

The application of chemical or thermal modification technique can be considered as a solution to this, since species of inferior properties, mainly softwoods, can be modified and transformed to entirely new green products with superior properties [4,5,6]. An interesting article recently reviewed the state of the art in the area of chemical and thermal modification. The article focused on two most promising techniques, which are acetylation and furfurylation [7].

The process of thermal modification successfully enhances wood’s ability to maintain its shape and protect against fungal attacks [8,9]. The reduction in mechanical properties limits the industrial applications in which the strength of wood is of prime importance. The improvement of dimensional stability and fungal resistance comes at the expense of reduced mechanical properties in this material. Thermal modification causes a known process which leads to the degradation of main wood components, which include cellulose and hemicellulose and lignin [3]. The process of thermal modification results in weight loss which increases when the temperature for thermal modification increases [10]. The heating process at temperatures below 200 °C causes some semi-crystalline cellulose areas to transform into crystalline material while cross-linking and polycondensation processes take place in lignin structure. The properties of wood receive substantial improvement from these factors, but the loss of weight shows only minimal evidence of cell wall polymer degradation [11,12].

The scientific field of nanotechnology offers an attractive research area which shows tremendous promise for developing new products that will exhibit superior performance characteristics [13]. The material properties undergo transformation because the interfacial surface area increases when particles reach their nanometer size limit. Nanomaterials improve the characteristics of base materials while maintaining strong compatibility with conventional materials and producing minimal changes to their base properties [14,15]. The purpose of using nanosized metals and minerals in wood products is to enhance both physical and mechanical attributes while extending resistance to microorganisms because it is widely understood that these materials contact bacterial elements which ultimately lead to cell death or disrupt enzyme activity [14,15,16,17]. The outer layer of wood products shows low thermal conductivity which makes it likely that different sections of wood products will undergo different thermal changes during thermal treatment [15,18]. The process of treating specimens with a silver nano-metal suspension will result in improved thermal conductivity which leads to equal thermal changes in both outer and inner specimen parts [19,20].

The above mentioned treatments have proven effective but pose environmental and health risks. As a result, there has been a significant interest in developing natural or less hazardous protective strategies. Among these, the use of mineral fillers, including silicate minerals, has gained attention due to their non-toxic nature and potential antifungal properties. Wollastonite, a naturally occurring silicate mineral, is one such material that has shown promise as a non-hazardous additive for enhancing wood properties. As an abundant, naturally occurring material, wollastonite offers a sustainable alternative to synthetic chemicals traditionally used in wood preservation. Its use in this context aligns with the increasing focus on developing environmentally friendly, sustainable materials in the wood industry. Studies have reported that wollastonite is safe for both human health and wildlife, making it an attractive alternative to traditional chemical preservatives [21,22].

In addition to its low toxicity, wollastonite has been shown to possess various beneficial effects. It has demonstrated the ability to enhance plant growth and exhibit inhibitory effects on certain pathogens, including fungi [23,24]. Recent research has explored its potential to improve the durability of wood and wood composites, with studies investigating its incorporation into resins and adhesives to enhance their antifungal properties [25,26,27]. Notably, the addition of nanowollastonite to wood composites has been found to improve the resistance of materials to fungal attack, making it a promising candidate for use in wood preservation [28,29].

Furthermore, wollastonite’s ability to be easily incorporated into acrylic-latex paints was also a key consideration in its selection. The mineral is known to be stable and easily dispersible in water-based paints, making it a practical candidate for surface applications. Its inclusion in paints can provide a convenient, simple, and effective method for applying antifungal protection to solid wood surfaces, without the need for complex or hazardous treatments.

Despite these findings, the application of wollastonite in surface coatings, specifically in paints designed to protect solid wood from fungal decay, remains largely unexplored. While the use of nanofillers in paints to improve fire resistance in wood has been investigated [29], no significant studies have addressed the potential of wollastonite-enhanced paints for protecting wood against biological deterioration caused by fungi such as *C. puteana*.

Acrylic resin-based paints have been widely applied as surface coatings to protect wood from fungal decay due to their ease of use, water resistance, and film-forming properties [30,31,32]. Studies have shown that acrylic coatings can provide a physical barrier against moisture and some microorganisms, but their effectiveness against aggressive brown-rot fungi like *Coniophora puteana* is limited, particularly on heat-treated softwoods. Despite these applications, there is a lack of research on enhancing acrylic paint performance with mineral additives, such as wollastonite, specifically for improving antifungal properties. This gap in the literature forms the basis of the present study.

*Coniophora puteana* was selected as the target microorganism due to its well-documented aggressiveness and widespread use as a model brown-rot fungus for evaluating wood decay and the effectiveness of protective coatings. This fungus rapidly degrades cellulose and hemicellulose in wood under high humidity conditions, making it a reliable indicator of antifungal performance [1,2,4]. Its extensive use in previous studies allows for comparison of results across different wood treatments and coating formulations. Understanding these degradation mechanisms provides a foundation for evaluating how wollastonite-enriched acrylic coatings may protect wood. Potential protective mechanisms include creating a physical barrier, modifying moisture dynamics in the wood, and potentially interfering with enzymatic activity, thereby reducing the rate and extent of fungal decay. This scientific rationale supports the experimental approach adopted in the present study. Therefore, testing the efficacy of wollastonite-enriched acrylic paint against *C. puteana* provides meaningful and standardized data on its potential to protect wood against one of the most challenging fungal species.

Wood-decaying fungi, such as *Coniophora puteana*, degrade wood primarily through enzymatic and oxidative mechanisms targeting the main structural polymers. Brown-rot fungi rapidly depolymerize cellulose and hemicellulose while partially modifying lignin, leaving a brittle, discolored lignin-rich matrix. The degradation involves secretion of cellulases, hemicellulases, and generation of reactive oxygen species, which collectively break down polysaccharide components of the cell wall, reduce mechanical strength, and lead to mass loss [2,3]. Enzymatic activity allows the fungus to penetrate cell walls, transport nutrients, and colonize the wood structure efficiently. In untreated specimens, this results in extensive cell wall breakdown, rapid deterioration of structural integrity, and visible decay. In treated specimens, such as those coated with acrylic paint or wollastonite-enriched paint, the fungal penetration is slowed or partially inhibited due to physical barriers, moisture limitation, and potential interactions between mineral particles and fungal enzymes [1,9,25]. Understanding these degradation mechanisms provides a basis for evaluating the protective effects of coatings and thermal modification, as the extent of decay depends on the ability of the fungus to access and degrade cellulose and hemicellulose in the wood.

Therefore, the present study addresses this gap by evaluating the antifungal efficacy of wollastonite-enriched acrylic paint on both unheated and heat-treated spruce wood. By incorporating wollastonite, we aim to improve fungal resistance beyond what conventional acrylic coatings can achieve, providing a simple, environmentally friendly, and effective surface treatment for solid wood.

Spruce (*Picea abies*) is one of the most widely used softwoods in construction, furniture production, and the manufacturing of musical instruments due to its favorable mechanical properties, light weight, and dimensional stability. Its straight grain and workability make it a preferred material in both industrial and artisanal applications. However, like other softwoods, spruce is highly susceptible to fungal decay, especially under high moisture conditions. Protecting spruce wood from biological degradation is therefore critical to maintaining its structural integrity, extending its service life, and reducing economic losses. Given its economic and functional importance, studies aiming to enhance the durability of spruce wood, including the use of thermal treatments, coatings, or mineral additives, have significant practical relevance. This makes spruce an ideal model species for evaluating the efficacy of protective strategies, such as the incorporation of wollastonite into acrylic paint, against aggressive wood-decaying fungi.

## 2. Materials and Methods

### 2.1. Specimen Preparation

Based on availability and industrial applications, spruce (*Picea abies* L. H. Karst.) lumbers were prepared from Tehran Wood Market (Tehran, Iran). Based on the seller’s report, the spruce lumbers were originally from Tver region (Russia). Their density was 0.44 g.cm^−3^.

Specimens of 20 × 20 × 50 mm size were prepared in longitudinal direction. Specimens were free from fungal or insect attack, cracks or checks, knots, or any other visual defects. Once the specimens were cut to size, thirty specimens were selected and randomly divided into six groups, allowing five replicate specimens for each group. Three groups were randomly arranged in a laboratory oven to be heat-treated at 185 °C for four hours. Narrow strips of plywood were put beneath specimens to avoid direct contact with the metal tray of the oven. Once heat-treated, they were kept in room conditions (25 ± 2 °C, 45 ± 3% relative humidity) for a month, along with unheated specimens.

### 2.2. Coating of Specimens

Each of the two main groups, unheated and heat-treated spruce wood, was divided into the following three sub-groups: (1) control (without any paint or wollastonite), (2) painted with plain acrylic paint, and (3) painted with acrylic paint containing wollastonite.

A water-based acrylic-latex paint (ALCO-6510, Alvan Paint and Resin Production Co., Tehran, Iran) was used in this study. The paint contained 37% ± 1% solid material. Two coats were applied by brush to each specimen, achieving a 210 ± 10 μm dry paint film on all surfaces. A 24 h interval was allowed between coats to ensure proper drying. After application, all specimens were conditioned at room temperature (25 ± 1 °C, 40% ± 2% relative humidity) for one month.

For the wollastonite-treated specimens, 15 wt% of wollastonite (based on the wet weight of the paint) was incorporated into the acrylic paint prior to application. The wollastonite suspension (60% solid content) was produced by Mehrabadi Manufacturing Company (Tehran, Iran), following the formulation reported by Taghiyari [29] (Table 1). To obtain this composition, dry wollastonite powder was gradually added to deionized water under continuous stirring to prevent aggregation. The mixture was homogenized using a high-shear mixer at 500 rpm for 20 min, producing a uniform suspension with stable dispersion. No additional dispersing agents were used. The resulting suspension was stored in sealed containers to prevent sedimentation and was briefly stirred before being incorporated into the acrylic paint. During mixing, the solid content of the final paint-wollastonite mixture was monitored and adjusted by adding distilled water as needed. Approximately 2.7 g of plain paint and 3.4 g of wollastonite-paint mixture were used per specimen. After coating, specimens were stored in a climate chamber at 25 ± 1 °C and 45 ± 1% relative humidity for two weeks.

### 2.3. Exposure to Coniophora puteana

The prepared specimens were exposed to the brown-rot fungus *Coniophora puteana*. All specimens were autoclaved at 121 °C for 20 min. The *C. puteana* (Schumach.) P. Karst. (brown cellar fungus) isolate 167 was derived from the strain collection of Olaf Schmidt (Hamburg, Germany). The *C. puteana* isolate was re-identified by rDNA-ITS sequencing.

Prior to incubation, actively growing mycelium from 7- to 10-day-old cultures was transferred to fresh malt extract agar plates and incubated for an additional 7 days to produce sufficient biomass. For fungal exposure, sterilized wood specimens were placed in contact with the actively growing mycelium in sterile Petri dishes or jars, ensuring full contact between the fungal culture and the wood surfaces. Specimens were exposed to the fungus in 500 mL household preserving jars on 100 mL malt agar (2% malt extract, Oxoid, 1.5% agar, Oxoid, Basingstoke, UK) when the agar was totally overgrown by mycelium. Parafilm was used to seal the preserving jars against moisture loss. All specimens were incubated for four months in a conditioned chamber in the University of Hamburg (Hamburg, Germany). Following incubation, mycelia on the surface of specimens were slightly removed so that the substrate was not distorted. The specimens were then dried at 103 °C for 24 h and weighed to determine the fungal mass loss.

Before any treatment or fungal exposure, all wood specimens were conditioned at 25 ± 1 °C and 45 ± 1% relative humidity for two weeks to reach equilibrium moisture content. Each specimen was then weighed using an analytical balance (±0.001 g) to determine the initial dry mass. These initial weights were recorded and used as the reference values for calculating mass loss after fungal exposure.

Following this, specimens were coated with either plain acrylic paint or wollastonite-enriched acrylic paint, as described in Section 2.2, and allowed to dry under controlled conditions prior to inoculation with *Coniophora puteana*.

### 2.4. Compression Strength Parallel-to-Grain

Compression strength parallel to grain test was carried out based on DIN 2-185 standard specifications, using a Zwick/Roell Z050 universal testing machine (Ulm, Germany) at Thuenen Institute of Wood Research, Braunschweig, Germany. The machine had a capacity of 50 kN load cell. Loading speed for all specimens was 0.8 mm.min^−1^. Compression strength values were calculated using the following equation:

$$P_{c||}=\frac{F_{\max}}{A}\;(N.mm^{-2})$$

where $P_{c||}$ is the compression strength parallel to grain (N/mm^2^), *F*_max_ is the maximum force (N), and *A* is the cross section of specimens (mm^2^).

### 2.5. Scanning Electron Microscopy

An FEI Quanta 250 FESEM (field-emission scanning electron microscopy, FEI Company, Hillsboro, OR, USA) was used. Initially, the specimens were cut to a size of 10 × 10 × 2 mm. The target surface was then carefully smoothed using a razor blade followed by being mounted onto aluminum stubs with carbon paste. They were then sputter-coated with thin layer of gold.

### 2.6. Statistical Analysis

A two-way analysis of variance (ANOVA) was carried out using SAS software, version 9.2 (2010) at a 95% level of confidence. Duncan multiple range test was then performed to determine groupings for each property by SPSS/18 software (2010). Contour and surface plots, in addition to calculation of correlation among mass losses versus compression parallel to grain, was carried out by Minitab software (version 16.2.2, 2010).

## 3. Results and Discussions

**Figure 1:**
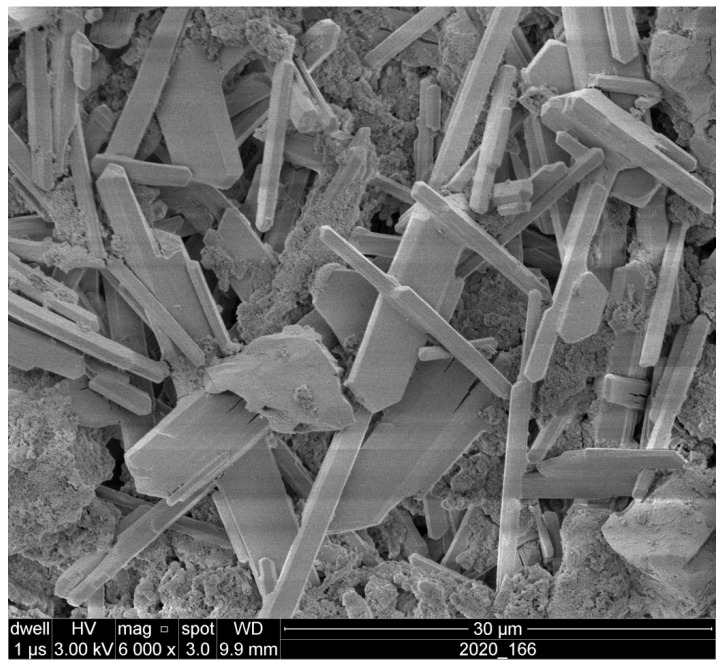
SEM image of wollastonite suspension.

Figure 1 shows the scanning electron microscope (SEM) image of wollastonite suspension, highlighting the morphology of the wollastonite particles at high magnification. As it can be seen, the wollastonite particles appear in needle-like or elongated forms, which are typical of this silicate mineral. The needle-like shape of the wollastonite particles is crucial as it influences the physical properties of the suspension when incorporated into the acrylic paint. The shape and size of the particles can affect the distribution of the material within the paint matrix and contribute to its effectiveness in providing antifungal protection. Furthermore, the elongated structure of the wollastonite particles also increases the surface area available for interaction with the wood surface, which may enhance the protective properties of the paint against fungal decay. A higher surface area can contribute to the adhesion of the particles to the wood and improve the overall durability of the coating.

### 3.1. Biological Durability

**Figure 2:**
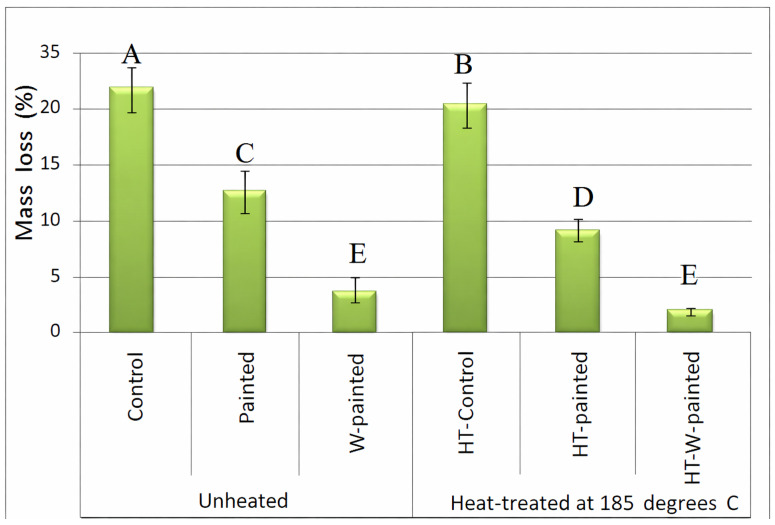
Mass losses (%) in spruce (*Picea abies*) wood specimens as a result of exposure to *Coniophora puteana* for four months in preserving jars (painted = painted specimens with plain acrylic paint; W-painted = wollastonite-added acrylic paint; HT = heat-treated spruce specimens) (letters on each column indicate the Duncan multiple range groupings at 95% level of confidence).

Figure 2 presents the control specimens, which exhibit the highest mass losses; that is, a mass loss of 23.8% was observed in the unheated and unpainted specimens. Mass loss in the heat-treated specimens was 20.2%, showing an improvement of only 15% in the biological resistance against *C. puteana*, though the improvement was statistically significant. This improving result was in agreement with previous reports, indicating the effects of heat treatment and thermal modification of solid wood species against wood decay fungi [3]. Specimens coated with plain paint demonstrated a high significant decrease in ML values. Still, addition of wollastonite to the paint resulted in an even more drastic decrease in ML compared to both control and painted specimens. ML values in all specimens coated with wollastonite-added paint were below 4% for both unheated and heat-treated specimens, demonstrating the high impact of wollastonite to protect spruce wood against *C. puteana*. The ML value in the unheated specimens which were coated by wollastonite-added paint decreased by more than 86% compared to the unpainted specimens (the control specimens). This clearly illustrated the great impact of wollastonite in protecting spruce wood against *C. puteana*.

In control specimens, which were uncoated and unmodified, *Coniophora puteana* actively degraded the wood by enzymatic hydrolysis of cellulose and hemicellulose, resulting in significant mass loss and reduction in mechanical strength. The absence of any protective coating allowed the fungus to penetrate the cell walls freely. In specimens coated with plain acrylic paint, the paint acted as a physical barrier, partially preventing fungal penetration and moisture absorption. As a result, the degradation was slower than in control samples, although some fungal growth occurred in areas with microcracks or incomplete coverage. In wollastonite-enriched acrylic paint specimens, the combination of the physical barrier provided by the paint and the presence of wollastonite particles further inhibited fungal colonization. The mineral particles may interfere with fungal enzymatic activity and modify surface moisture dynamics, resulting in significantly lower mass loss and higher retention of mechanical properties. Thermal treatment at 185 °C induced changes in the wood surface, including partial degradation of hemicellulose, increased crystallinity of cellulose, and chemical modifications in lignin. These changes decreased the hygroscopicity of the wood and limited fungal colonization. As observed in Figure 2, these surface modifications are consistent with improved fungal resistance, particularly in the wollastonite-treated, heat-modified specimens.

**Figure 3:**
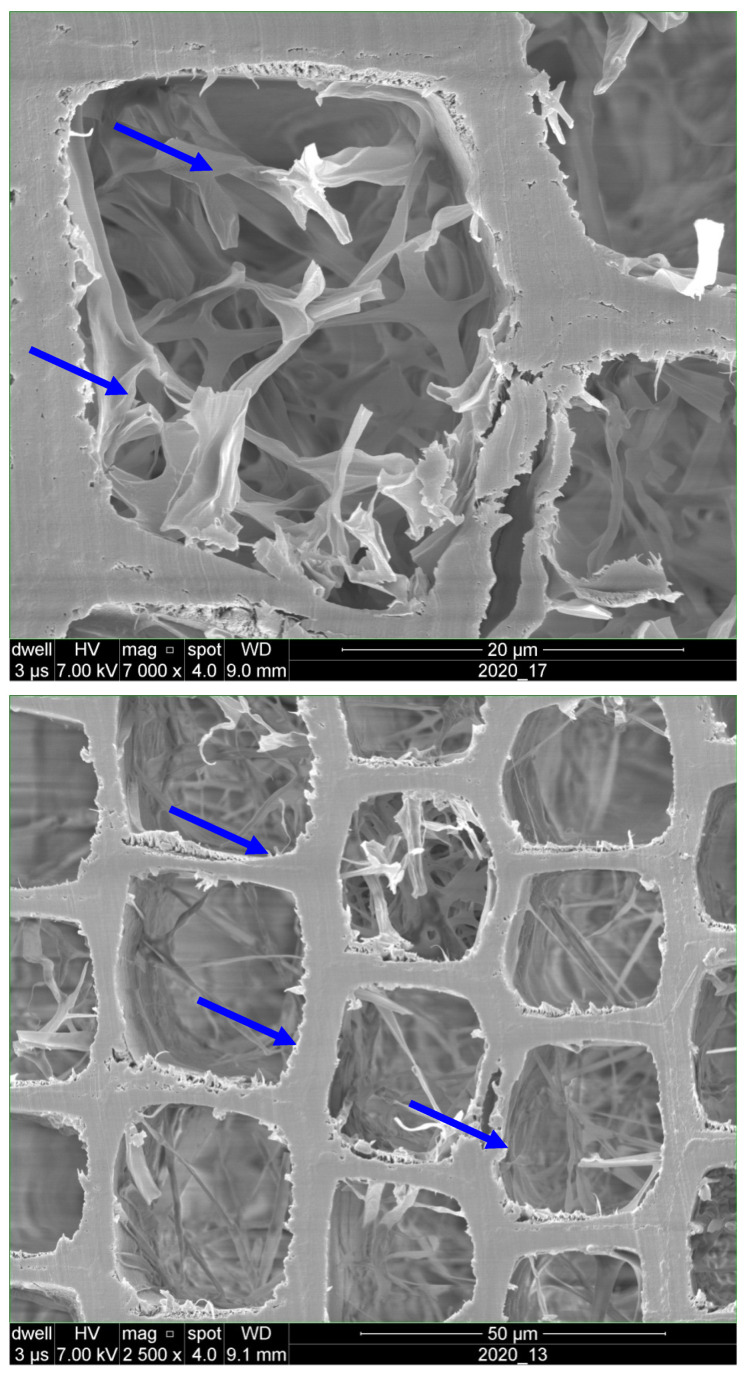
SEM images of spruce (*Picea abies*) specimens showing intense hyphae network within cell cavities (↓) in control specimens exposed to *Coniophora puteana* for four months.

SEM images of control specimens showed the growth of intense network of hyphae within cell lumen (Figure 3). Visual observation of specimens revealed severe decay in the control specimens exposed to *C. puteana* with iconic cubical patterns of checking.

In the present study, wollastonite acted in several ways as follows: (1) forming new bonds with the acrylic paint, chemically reinforcing the paint layer, (2) possibly forming bonds with the xylan on the surface of wood substrate, and (3) acting as a physically reinforcing filler in the paint layer. The combination of both chemical and physical reinforcements ultimately protected the wood substrate against the penetration of the fungal mycelium.

**Figure 4:**
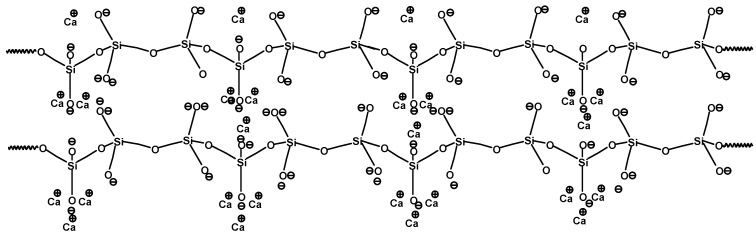
The chemical structure of the wollastonite chain with its load of coordinated Ca^++^ [3].

**Figure 5:**
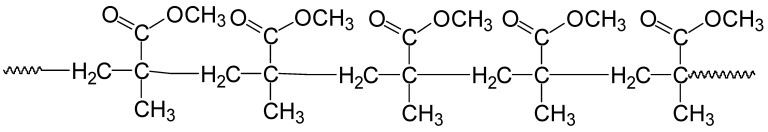
The chemical structure of the acrylic polymer chain [3].

The chemical structure of wollastonite and that of the acrylic polymer composing the resin are shown in Figure 4 and Figure 5. Based on the above two chemical structures (Figure 4 and Figure 5), the possible chemical interactions between wollastonite and acrylic paint can be multiple and of different types. Firstly, the ionic coordination of the positively charged Ca^2+^ of wollastonite linking to the negatively charged C=O of the methacrylic acid component of the paint creates strong new bonds, ultimately reinforcing the paint layer. The abundance of calcium ions, which is characteristic of the wollastonite structure, suggests that the formation of such potential ionic/coordination bonds can significantly contribute to the integrity of the paint layer. This interaction is consistent with previous studies that describe the formation of ionic bonds between metal cations and organic molecules in composite systems [31,32,33]. Additionally, secondary forces, such as electrostatic interactions and van der Waals forces, are likely to contribute to the overall stability of the paint coating. These forces have been widely recognized as crucial for enhancing the adhesion and mechanical properties of paint films when inorganic particles are incorporated [34,35].

Moreover, it is likely that ionic interactions were formed between the calcium ions of wollastonite and the oxygen atoms in the xylan of the wood substrate. At the same time, based on the chemical structure of methylglucuronoxylan presented by Fengel & Wegener [19], possible hydrogen bonds could be formed between the positively charged hydrogen atoms in the hydroxyl groups of xylan in the wood substrate and the charged oxygen atoms on the surface of wollastonite particles. There can also be numerous electrostatic and van der Waals interactions that can be formed, ultimately improving both the cohesion of the paint and its adhesion to the wood substrate. The extent and formation of the above mentioned chemical and ionic bonds can be the main topic of further research studied.

Apart from the chemical interactions and ionic coordination between wollastonite and acrylic paint molecules, wollastonite particles simultaneously acted as a physically reinforcing filler in the paint layer, prohibiting easy penetration of fungi mycelium through, eventually ameliorating severity of the fungal attack. Wollastonite was also reported to act as reinforcing filler in some resins, significantly increasing shear strength of the resins. Other materials, like graphene, acted as filler too, improving different properties including fire retardancy in beech [15].

### 3.2. Compression Strength Parallel to Grain

**Figure 6:**
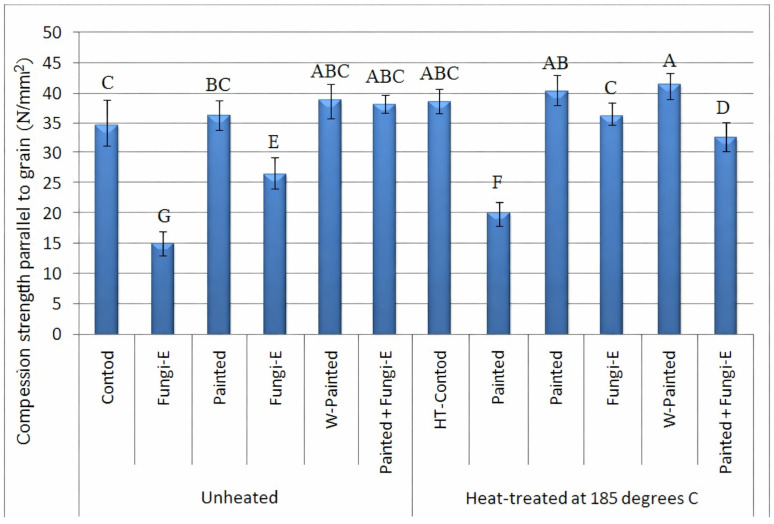
Compression strength parallel to grain (N/mm^2^) in spruce (*Picea abies*) specimens exposed to *Coniophora puteana* for four months in preserving jars (fungi-E = fungi-exposed; painted = painted specimens with plain acrylic paint; W-painted = wollastonite-added acrylic paint; HT = heat-treated spruce specimens) (letters on each column indicate the Duncan multiple range groupings at 95% level of confidence).

**Figure 7:**
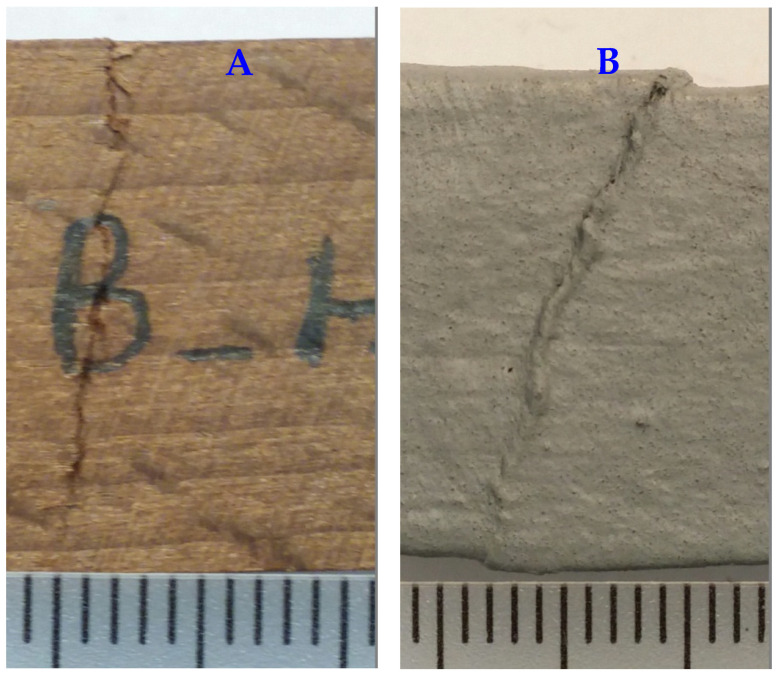

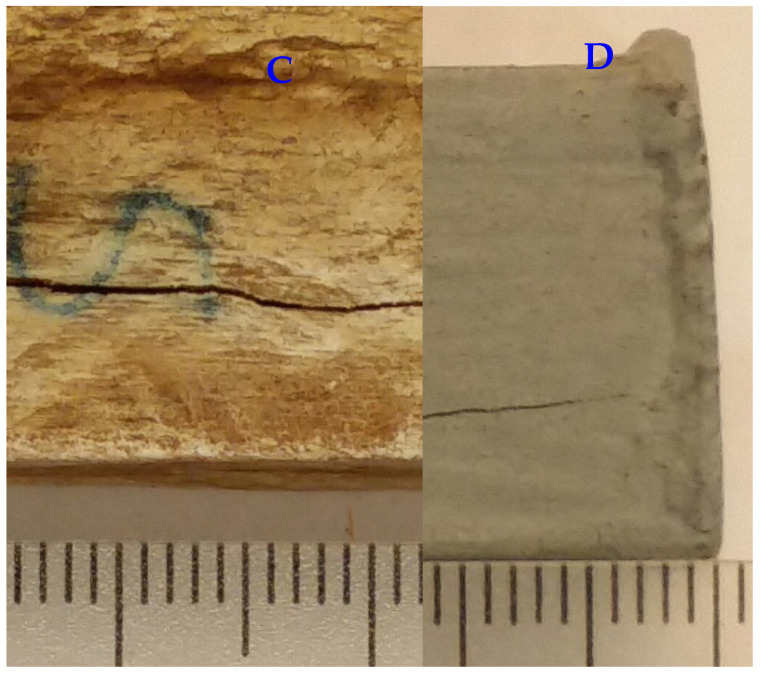
Modes of failure, including crushing (**A**) and shearing (**B**) in most of the unheated and heat-treated specimens, as well as splitting (**C**) and end-rolling (**D**) in most of fungi-exposed specimens (scales at the bottom of each photo are in mm).

Measurement of the compression strength parallel to grain demonstrated no significant difference between the unpainted with the painted (either plain or wollastonite-added) specimens, in either the unheated or heat-treated groups (Figure 6). This indicated that neither plain paint nor wollastonite-paint had any significant increasing effect on the compression strength, although the strength values in the painted specimen were slightly and insignificantly higher. Modes of failure in the unheated and heat-treated specimens were mostly either crushing (Figure 7A) or shearing (Figure 7B), though splitting was also observed in heat-treated specimens (Figure 7C). In the fungi-exposed specimens, modes of failure were mostly splitting (Figure 7C) or end-rolling (Figure 7D) or a mixture of all modes of failure.

The compression strength parallel to grain increased as a result of heat treatment. Heat treatment above the glass transition temperature of lignin, as seen in the current study, leads to the breakdown of covalent bonds between hemicellulose and lignin, forming reactive lignin fragments [36,37]. These fragments condense and repolymerize, creating new cross-links between lignin and hemicellulose, which enhances the overall strength of the wood structure [38]. Additionally, heat treatment induces hornification, a process where irreversible hydrogen bonds form within the wood’s cell walls, further contributing to the improved compression strength by increasing molecular chain mobility and rearrangement [39,40,41,42,43,44]. These combined effects of lignin repolymerization and hornification contribute to the increased compression strength observed in heat-treated specimens.

**Figure 8:**
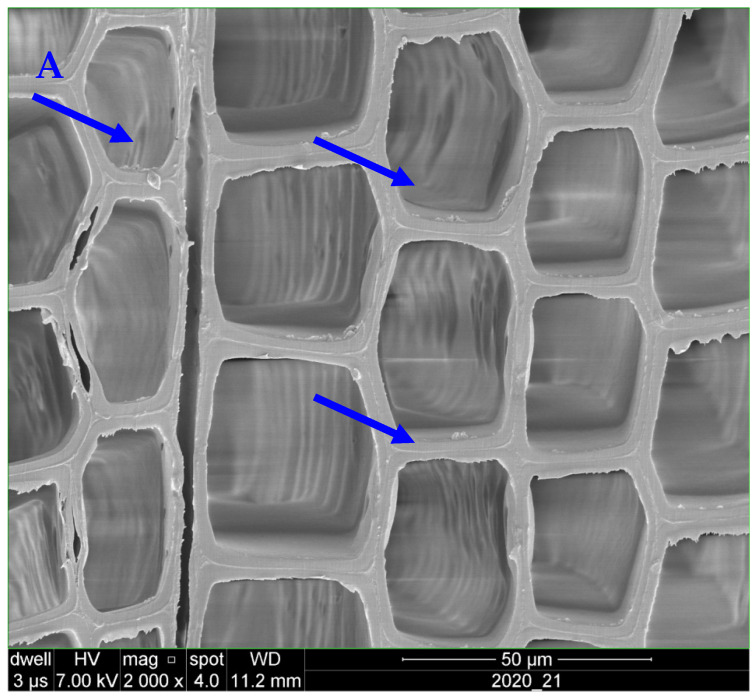

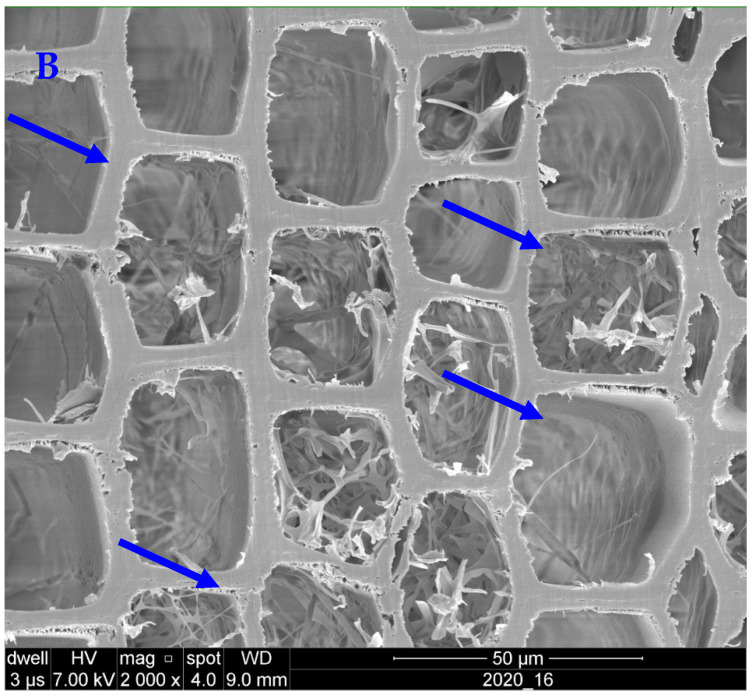
SEM images of the cross section of control spruce (*Picea abies*) specimens (**A**) showing complete and intact cell walls and empty lumen spaces (↓) and heat-treated specimen exposed to *Coniophora puteana* for four months (**B**) with deteriorated cell walls (↓).

Exposure to *C. puteana* had a drastically decreasing effect on the compression strength values in the control (unheated and unpainted) specimens; the values decreased in the control specimens by 65%. The SEM images (Figure 8A,B) highlight the effects of fungal degradation on wood structure, demonstrating the impact of *C. puteana* on the cell wall integrity, particularly in heat-treated specimens. SEM image (Figure 8A) showing the cross-section of untreated spruce (*Picea abies*) wood, highlighting the distinct cellular structure. The image clearly depicts the intact cell walls and the regular arrangement of the wood’s cellular components. The lumen spaces (empty areas) are visible within the cell cavities. This well-preserved structure serves as a baseline for comparison with treated specimens exposed to fungal degradation. SEM image (Figure 8B) showing the cross-section of spruce (*Picea abies*) wood exposed to *Coniophora puteana* fungal attack. The image highlights significant degradation within the wood’s cellular structure, with evident breakdown of the cell walls and the presence of fungal hyphal growth (visible in the lumen spaces). This damage illustrates the impact of fungal decay on wood integrity, providing a comparison to the untreated, intact wood structure shown in Figure 8B.

The SEM images clearly highlight the difference in the degree of fungal degradation between the untreated and heat-treated specimens. Heat treatment, while enhancing the wood’s resistance to fungal attack, does not completely prevent it. The changes observed in the cell walls, including the breakdown of structural components, correlate with the reduction in wood strength as indicated by the mass loss and compression strength measurements.

These findings provide valuable insight into the mechanisms behind the protective effect of wollastonite-added acrylic paint. The SEM analysis confirms that the treatment with the acrylic-wollastonite coating can help preserve the integrity of the wood structure by minimizing fungal colonization, as seen in the comparison with untreated and heat-treated specimens exposed to *C. puteana*.

Coating both unheated and heat-treated specimens with plain paint significantly ameliorated the negative effects of exposure to *Coniophora puteana*. This was evident from the reduced mass loss (ML) observed in the specimens coated with plain paint, compared to the uncoated controls, indicating a protective effect against fungal degradation. The addition of wollastonite to the paint demonstrated an even higher improvement in the protection of unheated specimens. As shown in the results, the specimens treated with wollastonite-enriched paint exhibited even lower mass loss values and better retention of compression strength when exposed to the fungus, particularly in the unheated specimens.

These data suggest that the incorporation of wollastonite into the acrylic paint formulation significantly enhances its ability to protect the wood from fungal degradation, even more effectively than the plain paint. Furthermore, the wollastonite coating offers a simple, cost-effective, and energy-efficient solution for preserving wood. The significant reduction in fungal degradation, as evidenced by the lower mass loss and maintained strength of the treated specimens, supports the potential of wollastonite-added paint to significantly extend the service life of wood in structural applications, thereby reducing the need for costly and energy-intensive wood replacements.

**Figure 9:**
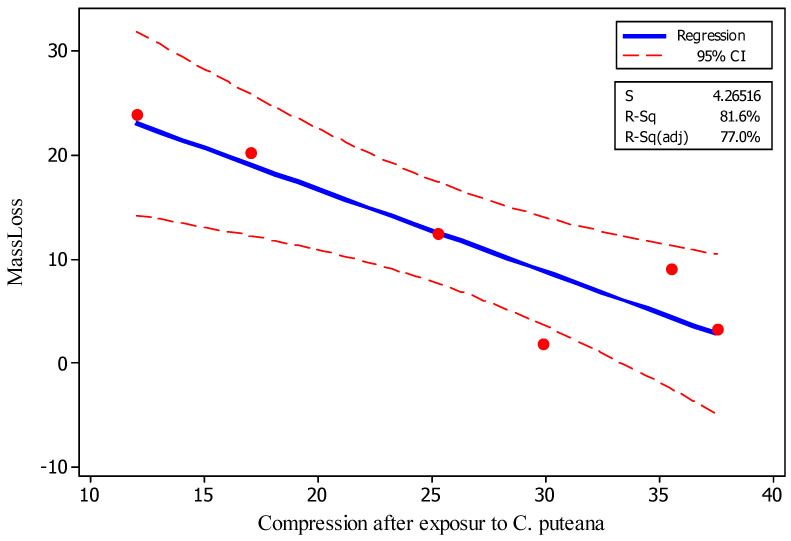
Fitted-line plot between mass losses versus compression strength parallel to grain values in spruce (*Picea abies*) specimens after exposure to *Coniophora puteana* for four months.

**Figure 10:**
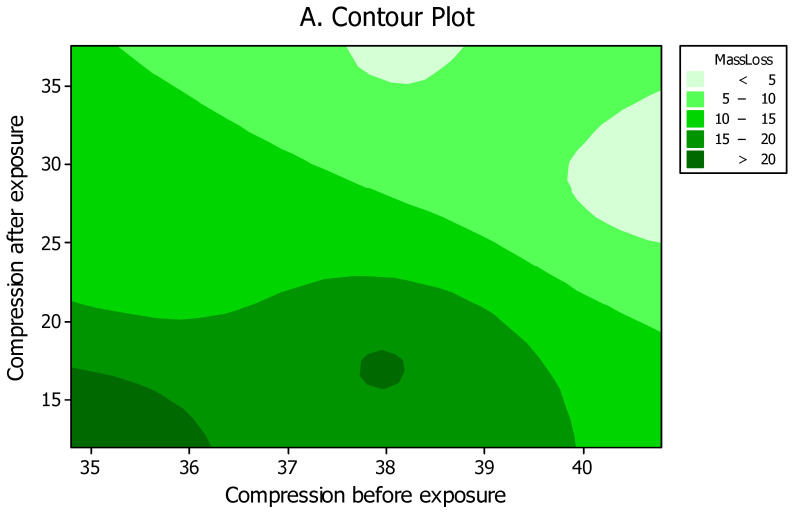

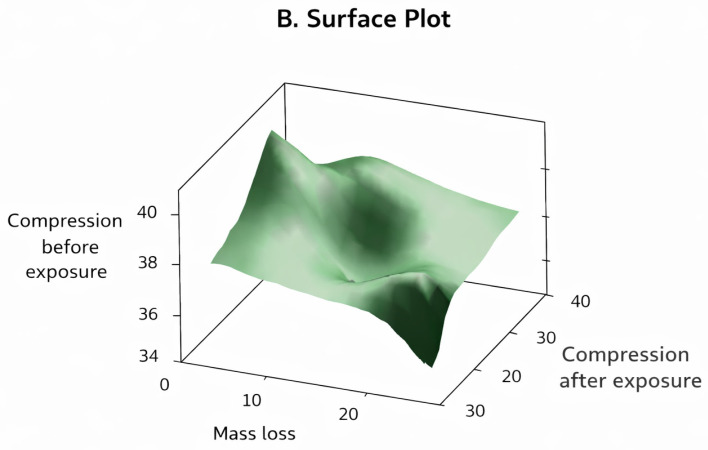
Contour (**A**) and surface (**B**) plots between mass losses versus compression strength parallel to grain values in spruce (*Picea abies*) specimens both before and after exposure to *Coniophora puteana* for four months.

High and statistically significant correlation (R-squared of 81.6%) was found between the mass losses and compression strength parallel to grain of specimens after being exposed to *C. puteana* (Figure 9). This clearly illustrated that compression strength can be directly linked to the degree of fungal deterioration. Contour and surface plots among the three properties of mass loss, compression strength parallel to grain both before and after being exposed to the fungus also demonstrated a clear trend among the properties (Figure 10A,B). Some slight discrepancies were observed in the trend which was attributed to the fluctuations and variances that exist in nearly all solid wood, as natural materials with high within-group variations.

**Figure 11:**
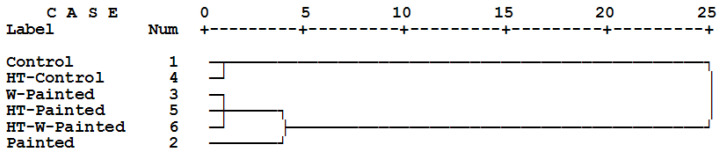
Cluster analysis for the five different treatments of spruce (*Picea abies*) specimens based on two properties of mass losses and compression strength parallel to grain values in spruce (*Picea abies*) specimens after being exposed to *Coniophora puteana* for four months.

Cluster analysis simultaneously carried out based on both mass loss and compression strength parallel to grain values of specimens after being exposed to *C. puteana* for four months revealed close clustering group of the unpainted specimens (control with heat-treated) (Figure 11). All painted specimens, regardless of being plain paint or wollastonite-added paint, were clustered very remotely from the unpainted specimens. This clearly proved the protecting effect of acrylic paint against the fungal attack. Among the four painted treatments, those specimens that were either heat-treated or in which wollastonite was added were closely clustered together. The specimens with plain acrylic paint were clustered a bit different from them. This indicated that both heat treatment and addition of wollastonite in the paint effectively improved the biological resistance of spruce wood against *C. puteana*.

## 4. Conclusions

The research establishes that wollastonite-enhanced acrylic paint functions as an effective environmentally safe method to shield spruce wood from damage caused by *Coniophora puteana*, which is a destructive brown-rot fungus. The protective performance of the paint system increased through the addition of wollastonite because the combination of these two elements produced superior defense mechanisms against unheated wooden materials. The enhancement occurs through chemical bonds between the wollastonite material and the paint which leads to stronger competition between these elements that results in higher durability for the wooden surface. Research results demonstrate that natural silicate minerals such as wollastonite create a novel method to substitute for traditional chemical-based wood preservatives. The wood protection method establishes a cost-efficient sustainable solution which extends the lifespan of wood materials across multiple use cases. The introduction of wollastonite into acrylic paint leads to energy-efficient wood preservation methods which reduce wood replacement needs and chemical treatment requirements while creating environmentally sustainable materials. This research establishes a framework for studying mineral-based coatings as protection methods for wood materials which scientists can use to research more effective coating solutions that function under varying environmental conditions.

In conclusion, *Coniophora puteana* degrades spruce wood primarily through enzymatic and oxidative breakdown of cellulose and hemicellulose, leaving a lignin-rich, brittle matrix and causing significant loss of mechanical strength. In untreated control specimens, this process led to extensive mass loss and structural deterioration. Application of plain acrylic paint provided a partial barrier, slowing fungal penetration and reducing mass loss, whereas the incorporation of wollastonite into the acrylic coating further inhibited fungal activity, likely by interfering with enzymatic action and modifying surface moisture dynamics. Thermal treatment at 185 °C also enhanced wood resistance by chemically modifying the wood polymers, reducing hygroscopicity, and limiting fungal colonization. These findings demonstrate that the combination of mineral-enriched coatings and thermal modification can effectively mitigate fungal degradation by targeting the mechanisms of wood decay.

## Figures and Tables

**Table 1:** Composition of the wollastonite suspension evaluated in the study [29].

Component	Proportion (% *w*/*w*)
SiO_2_	46.96
CaO	39.77
Al_2_O_3_	3.95
Fe_2_O_3_	2.79
TiO_2_	0.22
K_2_O	0.04
MgO	1.39
Na_2_O	0.16
SO_3_	0.05
Water	4.67

## Data Availability

The original contributions presented in this study are included in the article. Further inquiries can be directed to the corresponding authors.

## References

[1] Rowell R.M. (2012). Handbook of Wood Chemistry and Wood Composites.

[2] Gerardin P. (2016). New alternatives for wood preservation based on thermal and chemical modification of wood—A review. Ann. For. Sci..

[3] Hill C.A.S. (2006). Wood Modification—Chemical, Thermal and Other Processes.

[4] Papadopoulos A.N. (2010). Chemical modification of solid wood and wood raw materials for composites production with linear chain carboxylic acid anhydrides: A brief Review. BioResources.

[5] Mantanis G.I. (2017). Chemical modification of wood by acetylation or furfurylation: A review of the present scaled-up technologies. BioResources.

[6] Teng T., Arip M., Sudesh K., Lee H. (2018). Conventional technology and nanotechnology in wood preservation: A review. BioResources.

[7] Papadopoulos A.N., Bikiaris D.N., Mitropoulos A.C., Kyzas G.Z. (2019). Nanomaterials and chemical modification technologies for enhanced wood properties: A review. Nanomaterials.

[8] Boonstra M.J., Tjeerdsma B. (2006). Chemical analysis of heat treated softwoods. Holz Roh Werkst..

[9] Tjeerdsma B.F., Stevens M., Militz H. (2000). Durability Aspects of (Hydro) Thermal Treated Wood.

[10] Boonstra M.J., van Acker J., Tjeerdsma B.F., Kegel E.V. (2007). Strength properties of thermally modified softwoods and its relation to polymeric structural wood constituents. Ann. For. Sci..

[11] Tjeerdsma B.F., Boonstra M., Pizzi A., Tekely P., Militz H. (1998). Characterization of thermal modified wood: Molecular reasons for wood performance improvement. Holz Roh Werkst..

[12] Tjeerdsma B.F., Militz H. (2005). Chemical changes in hydrothermal treated wood: FTIR analysis of combined hydrothermal and dry heat-treated wood. Holz Roh Werkst..

[13] Roco M. (2006). Nanotechnology’s Future. Sci. Am..

[14] Taghiyari H.R., Schimdt O. (2014). Nanotachnology in wood based composite panels. Int. J. Bio-Inorg. Hybrid Nanomater..

[15] Goffredo G.B., Accoroni S., Totti T., Romagnoli T., Valentini L., Munafò P. (2017). Titanium dioxide based nanotreatments to inhibit microalgal fouling on building stone surfaces. Build. Environ..

[16] De Filpo G., Palermo A.M., Rachiele F., Nicoletta F.P. (2014). Preventing fungal growth in wood by titanium dioxide nanoparticles. Int. Biodeterior. Biodegrad..

[17] Civardi C., Schwarze F., Wick P. (2015). Micronized copper wood protection: An efficiency and potential health and risk assessment for copper based nanoparticles. Environ. Pollut..

[18] Moya R., Zuniga A., Berrocal A., Vega J. (2017). Effect of silver nanoparticles synthesized with NPsAg-ethylene glycol on brown decay and white decay fungi of nine tropical woods. J. Nanosci. Nanotechnol..

[19] Taghiyari H.R., Enayati A., Gholamiyan H. (2012). Effects of nano-silver impregnation on brittleness, physical and mechanical properties of heat-treated hardwoods. Wood Sci. Technol..

[20] Taghiyari H.R., Samandarpour A. (2015). Effects of nanosilver-impregnation and heat treatment on coating pull-off adhesion strength on solid wood. Drv. Ind..

[21] Huuskonen M.S., Jarvisalo J., Koskinen H., Nickels J., Rasanen J., Asp S. (1983). Preliminary results from a cohort of workers exposed to wollastonite in a Finish limestone quarry. Scand. J. Work. Environ. Health.

[22] Huuskonen M.S., Tossavainen A., Koskinen H., Zitting A., Korhonen O., Nickels J., Korhonen K., Vaaranen V. (1983). Wollastonite exposure and lung fibrosis. Environ. Res..

[23] Aitken E. (2010). Analyses of the Effect of Silicon on Fusarium Wilt on Banana.

[24] Whan J.A., Dann E.K., Aitken E.A.B. (2016). Effects of silicon treatment and inoculation with *Fusarium oxysporum* f. sp. *vasinfectum* on cellular defences in root tissues of two cotton cultivars. Ann. Bot..

[25] Papadopoulos A.N. (2020). Advances in wood composites. Polymers.

[26] Antov P., Lee S.H., Rhandi Lubis M.A., Yadav S.M. (2023). Potential of nanomaterials in bio-based wood adhesives: An overview. Emerging Nanomaterials.

[27] Savov V. (2023). Nanomaterials to improve properties in wood-based composite panels. Emerging Nanomaterials.

[28] Hassani V., Taghiyari H.R., Schmidt O., Maleki S., Papadopoulos A.N. (2019). Mechanical and physical properties of oriented strand lumber (OSL): The effect of fortification level of nanowollastonite on UF resin. Polymers.

[29] Taghiyari H.R., Bari E., Schmidt O. (2014). Effects of nanowollastonite on biological resistance of medium-density fiberboard against *Antrodia vaillantii*. Eur. J. Wood Wood Prod..

[30] Yuningsih I., Sekartining Rahayu I., Lumongga D., Darmawan W. (2019). Wettability and adherence of acrylic paints on long and short rotation teaks. Wood Mater. Sci. Eng..

[31] Bustamante-Torres M., Romero-Fierro D., Arcentales-Vera B., Pardo S., Bucio E. (2021). Interaction Between Filler and Polymeric Matrix in Nanocomposites: Magnetic Approach and Applications. Polymers.

[32] Kenny J.M., Nicolais L. (1989). Science and Technology of Polymer Composites. Comprehensive Polymer Science and Supplements.

[33] Hagita K., Morita H., Takano H. (2016). Molecular Dynamics Simulation Study of a Fracture of Filler-Filled Polymer Nanocomposites. Polymer.

[34] Smith J.S., Bedrov D., Smith G.D. (2003). A Molecular Dynamics Simulation Study of Nanoparticle Interactions in a Model Polymer-Nanoparticle Composite. Compos. Sci. Technol..

[35] Kickelbick G. (2003). Concepts for the Incorporation of Inorganic Building Blocks into Organic Polymers on a Nanoscale. Prog. Polym. Sci..

[36] Hatakeyama T., Nakamura K., Htakeyama H. (1982). Studies on heat capacity of cellulose and lignin by differential scanning calorimetry. Polymer.

[37] Phuong L.X., Shida S., Saito Y. (2007). Effects of heat treatment on brittleness of *Styrax tonkinensis* wood. J. Wood Sci..

[38] Weiland J., Guyonnet R. (2003). Study of chemical modifications and fungi degradation of thermally modified wood using DRIFT spectroscopy. Holz Roh Werkst..

[39] Borrega M., Kärenlampi P.P. (2010). Hygroscopicity of Heat-Treated Norway Spruce (*Picea abies*) wood. Eur. J. Wood Wood Prod..

[40] Fahlén J., Salmén L. (2003). Cross-sectional structure of the secondary wall of wood fibers as affected by processing. J. Mater. Sci..

[41] Hakkou M., Pétrissans M., Zoulalian A., Gérardin P. (2005). Investigation of wood wettability changes during heat treatment on the basis of chemical analysis. Polym. Degrad. Stab..

[42] Kato K.L., Cameron R.E. (1999). Structure-property relationships in thermally aged cellulose fibers and paper. J. Appl. Polym. Sci..

[43] Matsuda Y., Isogai A., Onabe F. (1994). Effects of thermal and hydrothermal treatments on the reswelling capabilities of pulps and paper sheets. J. Pulp Pap. Sci..

[44] Weise U. (1998). Hornification: Mechanisms and terminology. Pap. Timber.

